# Postural control of Parkinson’s disease: A visualized analysis based on Citespace knowledge graph

**DOI:** 10.3389/fnagi.2023.1136177

**Published:** 2023-03-24

**Authors:** Yan Li, Jie-Jiao Zheng, Xie Wu, Wen Gao, Chan-Jing Liu

**Affiliations:** ^1^Department of Rehabilitation Medicine, Huadong Hospital, Fudan University, Shanghai, China; ^2^Department of Sport Rehabilitation, Shanghai University of Sport, Shanghai, China; ^3^Shanghai Clinical Research Center for Rehabilitation Medicine, Shanghai, China

**Keywords:** Parkinson’s disease, postural control, postural balance (MeSH), Citespace, visualization, bibliometrics, knowledge graph

## Abstract

Postural control impairment is one of the primary motor symptoms in patients with Parkinson’s disease, leading to an increased risk of falling. Several studies have been conducted on postural control disorders in Parkinson’s disease patients, but no relevant bibliometric analysis has been found. In this paper, the Web of Science Core Collection database was searched for 1,295 relevant papers on postural control in Parkinson’s disease patients from December 2011 to December 2021. Based on the Citespace knowledge graph, these relevant papers over the last decade were analyzed from the perspectives of annual publication volume, countries and institutes cooperation, authors cooperation, dual-map overlay of journals, co-citation literature, and keywords. The purpose of this study was to explore the current research status, research hotspots, and frontiers in this field, and to provide a reference for further promoting the research on postural control in Parkinson’s disease patients.

## 1. Introduction

Parkinson’s disease (PD) is the second most common neurodegenerative disease after Alzheimer’s disease (Reich and Savitt, 2019; Talaat et al., 2021). As an age-related disease, the prevalence of PD rises with age (Tysnes and Storstein, 2017). In most cases, the etiology of PD is not clear, and it is thought to be the result of a combination of genetic and environmental factors (Kalia and Lang, 2015; Tysnes and Storstein, 2017). The main pathological features of PD are the loss of dopaminergic neurons in the Substantia Nigra compacta (SNpc) and the appearance of Lewy bodies and Lewy neurites composed of abnormally aggregated α-synuclein (Kalia and Lang, 2015), which leads to dyskinesia with typical motor symptoms (Radhakrishnan and Goyal, 2018). A variety of clinical symptoms are frequently present in PD patients. The clinical diagnosis of PD is based on the presence of motor symptoms (MS), including bradykinesia, myotonia, and resting tremor (Tysnes and Storstein, 2017; Cabeleira et al., 2019). Postural control impairment is a late motor feature in PD patients (Kalia and Lang, 2015). Freezing of gait (FOG) is a clinical phenomenon in which patients have a transient, episodic inability to stride or minimal stride length when starting to walk or turn (Nutt et al., 2011; Schlenstedt et al., 2016). According to studies, falls and freezing of gait occur in 80% of PD patients within 15 years of diagnosis (Hely et al., 2005). FOG and postural instability interact with each other (Bekkers et al., 2018a), and individuals with PD who have FOG have worse postural control and are more likely to fall (Coelho et al., 2021).

Postural control is a complex skill that involves the interaction of sensory systems, including somatosensory, vestibular, and visual, with motor processes to maintain the body in a spatial position as well as establish stability and orientation (Horak, 2006). Postural control keeps the gravity line within the base of support through anticipatory and/or compensatory adjustment strategies to prevent postural instability and falls (Romero and Stelmach, 2003). However, because the gravity line of PD patients tends to oscillate the medial and lateral direction of the support base, they are unable to perform compensatory movements to keep body balance, leading to an increased fear of falling (FOF; Gray and Hildebrand, 2000). FOF is an independent risk factor when assessing postural instability in individuals with PD, altering postural control in patients (Adkin et al., 2003). Impaired postural control increases the risk of falling in PD patients by increasing body sway, postural instability, and impaired coordination (Paul et al., 2014). Studies have shown that PD patients have 2–3 times more falls than healthy older adults (Pickering et al., 2007). As the disease advances, postural instability will deteriorate and negatively impact the quality of life (Debu et al., 2018). Therefore, studies on postural control in PD patients play an important role in improving postural instability and balance deficits and decreasing the risk of falls in PD patients.

As public awareness of Parkinson’s disease grows, the number of related documents has been increasing. There are some literature reviews on postural control in PD patients, but the traditional literature reviews generally have the disadvantages of low information utilization and small coverage. Therefore, a simple, efficient, and scientific method of bibliometric analysis has been discovered (Schlenstedt et al., 2016; Barbieri et al., 2019). The bibliometric analysis mainly relies on bibliographic databases, such as Scopus, PubMed, Web of Science (WOS), Cochrane Library, Embase, etc. (Luo et al., 2021). Compared to other databases, WOS provides a better knowledge graph for visualized analysis of Citespace (Falagas et al., 2008; Martín-Martín et al., 2021). Citespace is a Java-based information data analysis and visualization tool that investigates the evolution and frontiers of a scientific field by quantifying and visualizing the literature or collection of literature in that field (de Castilhos Ghisi et al., 2020; Zhong et al., 2020a). Citespace has currently been widely used by researchers in many research fields to explore research hotspots and frontiers in this field. However, there is still no information on Parkinson’s disease and visualized analysis in the field of postural control. Therefore, based on the Citespace software, taking the relevant literature retrieved from the WOSCC database as the data source, this paper conducts a visualized analysis of the annual volume of articles, international and institutional cooperation, authors cooperation, dual-map overlay of journals, literature co-citation, and keywords in this field, to explore the current research status, hot content and development trend of postural control research in PD patients in recent 10 years.

## 2. Materials and methods

### 2.1. Data source and retrieval strategy

All data were collected from the Web of Science Core Collection (WOSCC). Citation indexes were selected from the WOSCC including the Science Citation Index Expanded (SCI-Expanded), the Social Science Citation Index (SSCI), Current Chemical Reactions (CCR-EXPANDED), and Index Chemicus (IC) for advanced search. The retrieval term is “[TS = (Parkinson’s disease) OR TS = (Parkinson)] AND [TS = (postural control) OR TS = (postural balance)].” The retrieval time span is from 2011-12-30 to 2021-12-30. A total of 1,347 original documents were retrieved, including 64,631 references.

#### 2.1.1. Literature inclusion criteria

(1) The types of articles are mainly “article” and “review,” including early access, proceeding paper, book chapter, etc.; (2) The article was published from 2011-12-30 to 2021-12-30; and (3) All document languages are English.

#### 2.1.2. Literature exclusion criteria

(1) Meeting abstract; (2) Letter; (3) Editorial material; and (4) Correction.

### 2.2. Data conversion and processing

The retrieved original documents were exported in a plain text file, and the recorded contents included full records and cited references. The exported documents were named “download_XXX.txt” and then imported into Citespace 6.1 R1 software for document filtering and deduplication. The most common types of literature were selected as “article” and “review.” A total of 1,295 valid literature records were obtained, including 1,277 DOI documents, 1,103 “articles” (including eight early access and 17 proceedings papers), and 174 “reviews” (including two book chapters) respectively, while five editorial materials, two letters, and 45 meeting abstracts were excluded. A total of five editorial materials, two letters, and 45 meeting abstracts were excluded from the visualized analysis.

### 2.3. Analysis tools and methods

Citespace 6.1 R1 software was used as the visualized analysis tool. The time slice was from December 2011 to December 2021. The time slice was chosen as 1 year. “Top N = 50” denotes the top 50 cited documents for each extracted time slice (Xu et al., 2021). And the top N % is 10%.

### 2.4. Main outcome measures

**Figure 1:**
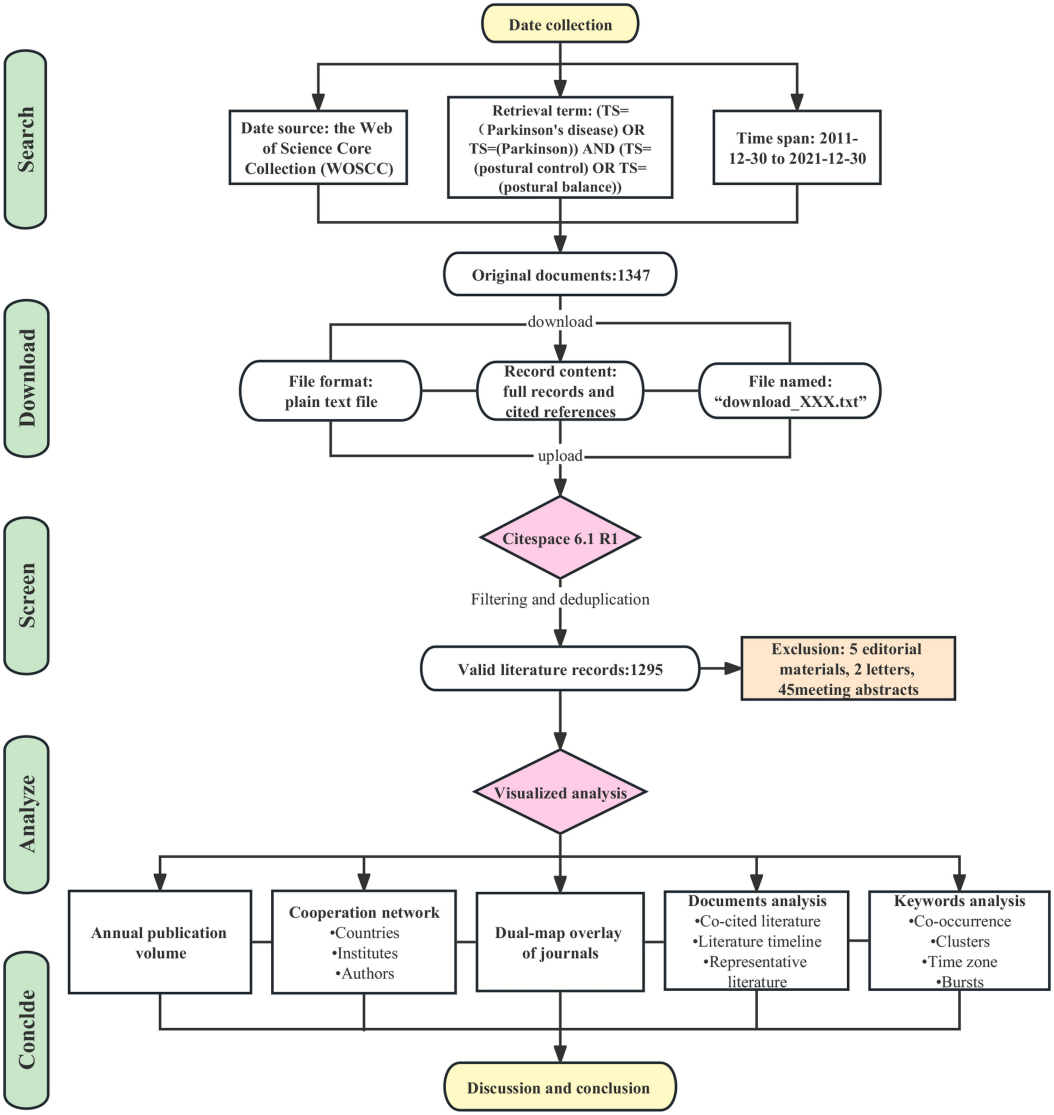
Flow chart of the study design.

(1) The analysis of annual publication volume; (2) The analysis of cooperation network among countries, institutes, authors, and dual-map overlay of journals; (3) The analysis of cited literature mainly contains network map, timeline map, high-frequency co-cited literature; and (4) Keywords analysis mainly involves keywords co-occurrence analysis, keywords cluster analysis, keywords time zone map and keywords burst analysis. The flow chart of the study design is shown in Figure 1.

## 3. Results

### 3.1. Analysis of annual publication volume

**Figure 2:**
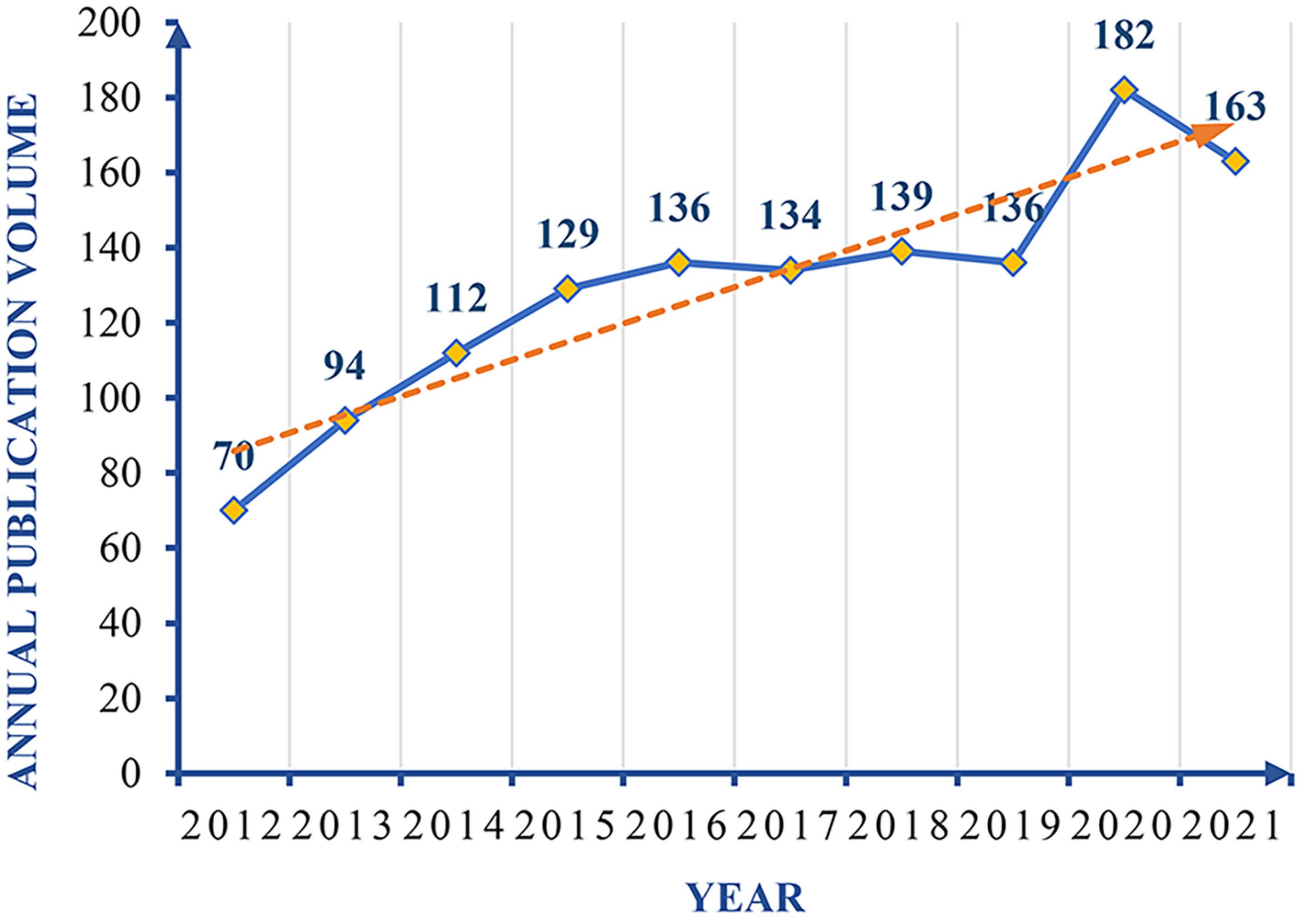
Trend chart of annual publication volume (2012–2021). Dotted lines represent trend lines.

In the recent 10 years, the number of articles published in the field of postural control of PD patients in the world has generally shown a wave-like upward trend (Figure 2). During the two periods of 2012–2016 and 2019–2020, the growth rate of the number of published articles continued to rise, and the number of published articles reached the highest in 2020 (182 articles). From 2016 to 2019, the annual number of posts has remained stable, but the trend line is generally on the rise. Over the increase years, the number of articles published in the research field of postural control of PD patients is increasing. It is seen that the research on postural control of Parkinson’s patients has gradually attracted the attention of international researchers, and may become a potential research hotspot in the future.

### 3.2. Analysis of countries and institutes cooperation network map

**Figure 3:**
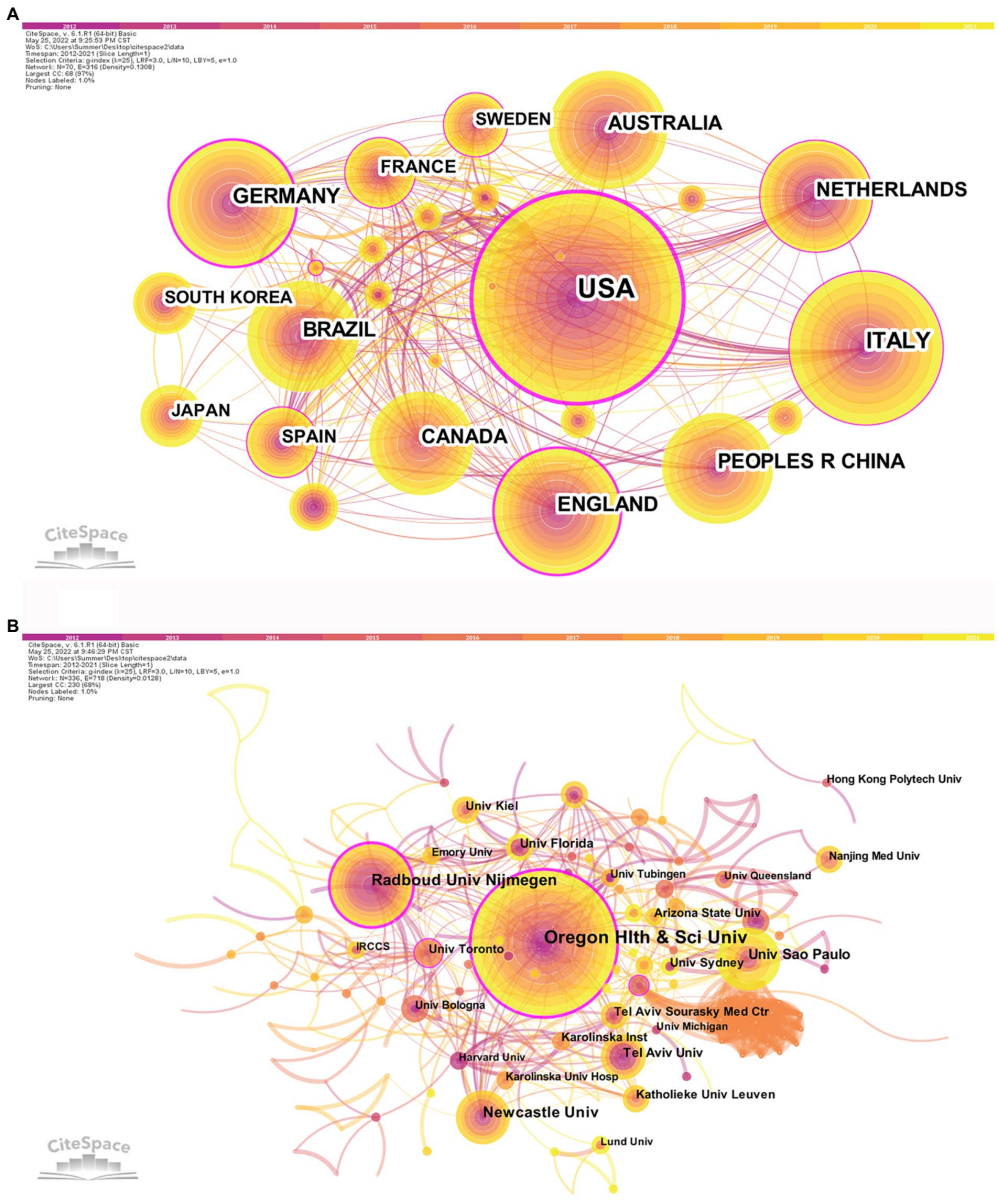
Countries **(A)** and institutes **(B)** cooperation network map of relevant literature. The colored bar at the top represents the year, with the years rising from left to right. The published volume by countries/institutes is indicated by the size of the circle. The lines separating the circles represent the international collaboration between countries/institutes. The more connections, the closer cooperation with other countries/institutions. The purple outer ring represents the betweenness centrality, and the thicker the purple outer ring means the greater the betweenness centrality.

After visualized analysis with “country” and “institute” as nodes, the countries and institutes cooperation network maps were obtained (Figure 3). A total of 70 countries have participated in postural control studies in patients with PD in the past 10 years, and there is closer cooperation between countries. The top three countries with the largest number of papers are the United States (407 papers, 31.43%), Italy (169 papers, 13.05%), and Germany (116 papers, 8.96%). The purple circle outside the circle represents the betweenness centrality (Wu et al., 2021). Betweenness centrality is used to measure the likelihood of any shortest path through a node in the network graph, which can evaluate the importance of each node in the graph. When the value of betweenness centrality is greater than 0.1, a node is considered critical (de Castilhos Ghisi et al., 2020). Among the top 10 countries in terms of the number of articles issued, the United States had the highest betweenness centrality (0.46), indicating the pivotal position of the United States in this research area (Table 1). The top three institutes with the highest number of publications were *Oregon Health and Science University* (75), *Radboud University Nijmegen* (45), and *Newcastle University* (34) in England. Among the top 10 publishing institutes, *Oregon Health and Science University* in the United States (0.29), *Radboud University Nijmegen* in the Netherlands (0.22), and the *University of Toronto* in Canada (0.12) have high centrality, indicating the high influence of the above three universities within the field of postural control in PD patients.

**Table 1:** Top 10 countries and institutions in the field of postural control in PD patients among 1,295 included studies (2011–2021).

Rank	Country	Count	Centrality	Institute (Country)	Count	Centrality
1	USA	407	0.46	Oregon Health and Science University (United States)	75	0.29
2	Italy	169	0.17	Radboud University Nijmegen (Netherlands)	45	0.22
3	Germany	116	0.36	Newcastle University (England)	34	0.08
4	England	105	0.24	University of Sao Paulo (Brazil)	32	0.04
5	China	93	0.01	Tel Aviv University (Israel)	22	0.05
6	Brazil	92	0.02	University of Florida (USA)	22	0.06
7	Netherlands	86	0.14	Arizona State University (United States)	18	0.04
8	Canada	82	0.08	University of Toronto (Canada)	18	0.12
9	Australia	79	0.1	University of Kiel (Germany)	18	0.05
10	France	50	0.18	Katholieke University Leuven (Belgium)	17	0.03

### 3.3. Analysis of authors cooperation network map

**Figure 4:**
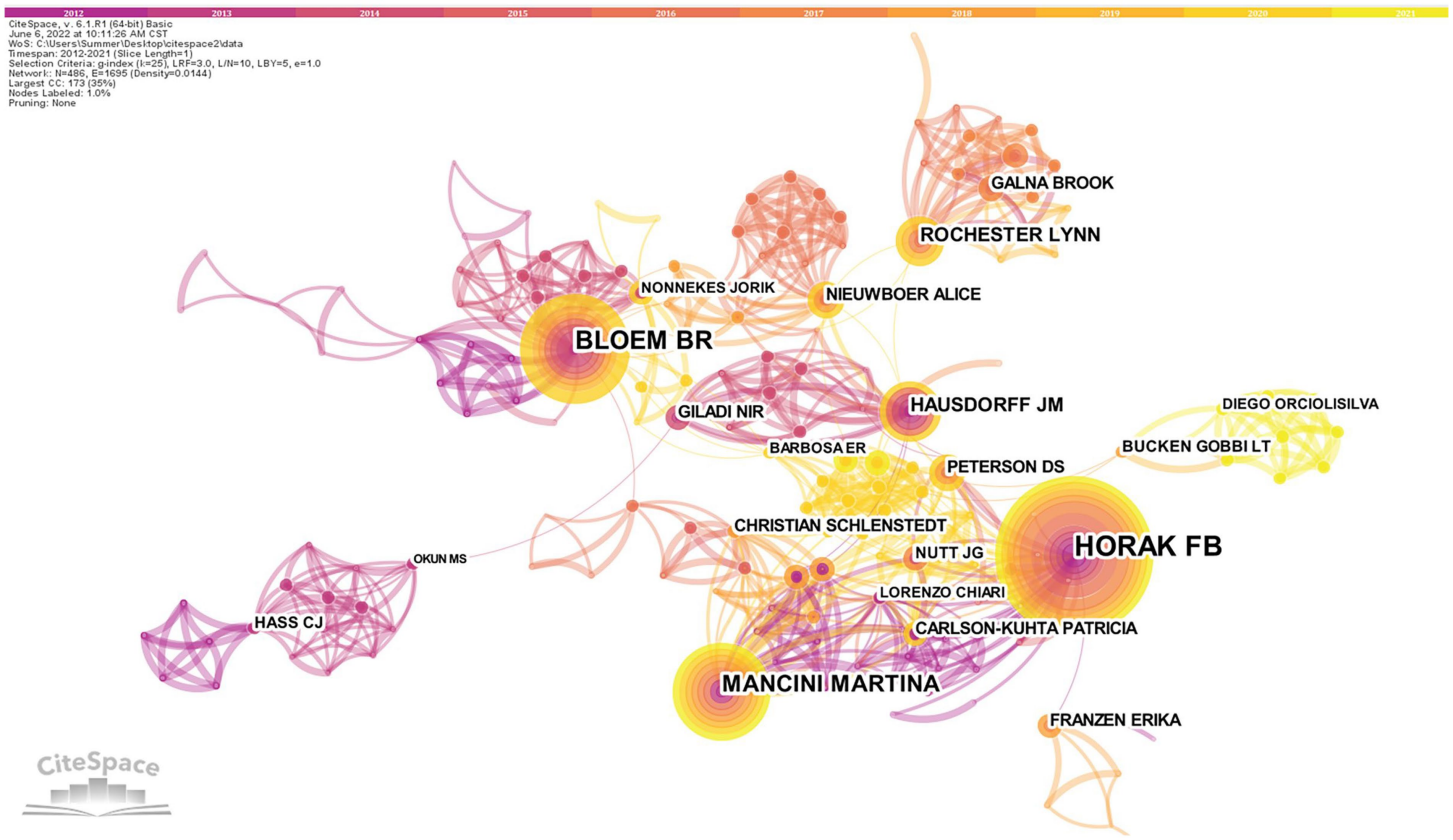
Authors cooperation network map of related literature. The size of the circle indicates how many articles the author has posted, and the circles represent the authors. The lines between the circles represent the cooperation of the authors. The tighter the cooperation with other authors, the more lines.

A total of 486 nodes and 1,695 lines of the authors cooperation network map were obtained after visualized analysis with “author” as the node (Figure 4). The top 10 authors in terms of the number of publications were selected by the number of publications (Table 2). The highest number of publications in the field of postural control research in PD patients in the last decade was Horak from Oregon Health and Science University, with 61 articles published. Bloem, from *Radboud University Nijmegen* (Netherlands), is second-ranked with 42 publications, and its high centrality (0.10) indicates the unique contribution of this scholar in this research area. The third-ranked Mancini, who also comes from *Oregon Health and Science University* (USA), is part of the same institute as the first-ranked Horak, and there is a close collaboration between them. There is an international academic team with Horak and Mancini as the core, but cross-site and cross-institutional cooperation between the authors is not evident.

**Table 2:** Top 10 authors who published literature on postural control studies in PD patients among 1,295 included studies (2011–2021).

Rank	Author	Institute	Count	Centrality
1	Horak, Fay B.	Oregon Health and Science University	61	0.08
2	Bloem, Bastiaan R.	Radboud University Nijmegen	42	0.10
3	Mancini, Martina	Oregon Health and Science University	33	0.01
4	Rochester, Lynn	Newcastle University	17	0.04
5	Hausdorff, Jeffrey M.	Tel Aviv University	15	0.05
6	Carlson-Kuhta, Patricia	Oregon Health and Science University	12	0.00
7	Giladi, Nir	Tel Aviv University	12	0.05
8	Galna, Brook	Newcastle University	12	0.00
9	Schlenstedt, Christian	University of Kiel	12	0.01
10	Earhart, Gammon M.	Washington State University	11	0.02

### 3.4. Visualized analysis of dual-map overlay of journals

The dual-map overlay of journals shows the position of research on the topic relative to the main research disciplines, obtains the flow of information at the journal level, and can visually reflect the research dynamics of the discipline. The dual-map overlay of journals consists of two main parts, with the citing journals on the left and the cited journals on the right. The curve between them is the citation route (Chen et al., 2014). Among the cited journals in the fields of psychology/education/social, *Movement Disorders* (IF = 9.698) was the most cited journal.

**Figure 5:**
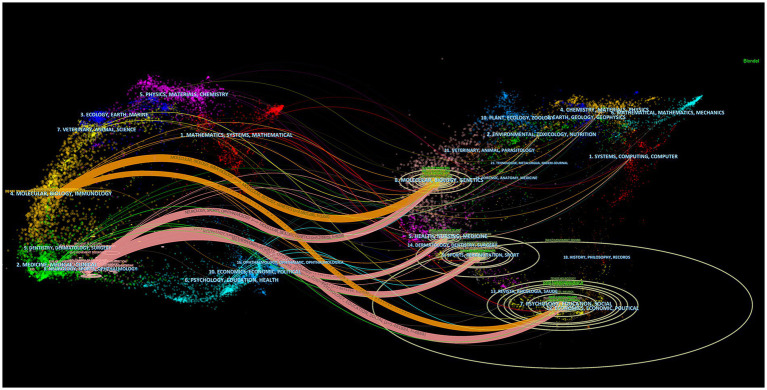
Dual-map overlay of journals that published literature on postural control of PD patients from 2011 to 2021. The ellipse in the figure represents the number of publications corresponding to a journal. The longer the horizontal axis of the ellipse is, the more authors are represented, and the longer the vertical axis of the ellipse is, the more papers are published on behalf of journals.

There are six main citation paths (Figure 5). The orange paths show that journals published in the fields of molecular/biology/immunology are usually influenced by journals published in the fields of molecular/biology/genetics (*z* = 3.6801286, *f* = 3,358) and psychology/education/social (*z* = 2.0435498, *f* = 1994). The pink paths indicate that journals published in the fields of neurology/sports/ophthalmology are typically influenced by journals published in the fields of psychology/education/social (*z* = 5.491884, *f* = 4,868), molecular/biology/genetics (*z* = 5.239918, *f* = 4,658), sports/rehabilitation/sport (*z* = 2.9674249, *f* = 2,764), and health/nursing/medicine (*z* = 2.2691193, *f* = 2,182). The dual-map overlay of journals predicts that hot spots and trends of postural control in PD patients will converge in the fields of neurology/sports/ophthalmology.

### 3.5. Documents analysis

#### 3.5.1. Co-cited literature analysis

**Figure 6:**
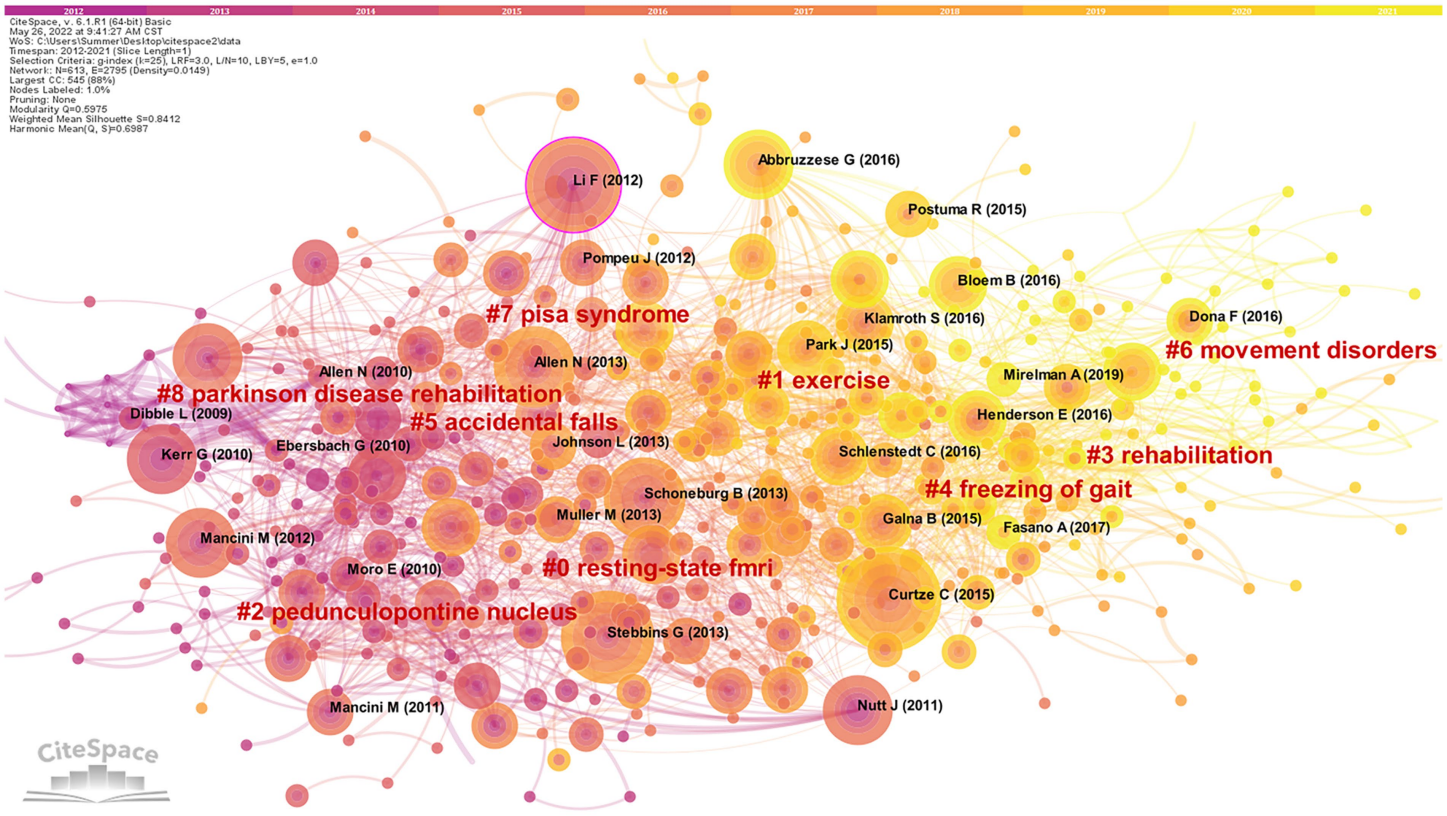
Cluster network map of co-cited literature. 613 notes and 2,795 connection lines emerged. Nodes of the same color represent the same cluster.

When two or more papers are cited by another paper, links are established between the cited and co-cited relationships in the literature, and this is referred to as co-citation (Wang et al., 2021). The visualized map generated with “reference” as the node can get 613 nodes, and 2,795 connected documents are cited in the network (Figure 6).

Nine groups of cluster labels (#0–8) were generated after clustering analysis of cited literature by using the Log-likelihood ratio (LLR). Each cluster silhouette is higher than 0.8, indicating that the research problem is narrow and the overall cluster effect is quite good. Among them, the maximum cluster label (#0) resting-state fMRI (84) is a neuroimaging technique. Clinically, resting-state fMRI is utilized to identify the functional connectivity of brain networks and cortical activity in PD patients (Mohl et al., 2017; Shen et al., 2017; Kaut et al., 2020), which provides new ideas and clues for the research of postural control mechanisms and the diagnosis of PD. The most recent cluster label for the referenced year is (#6) dyskinesia, indicating that the relationship between postural control and dyskinesia related to postural sway, freezing of gait, and falls are hot topics in recent years.

#### 3.5.2. Co-cited literature timeline map

**Figure 7:**
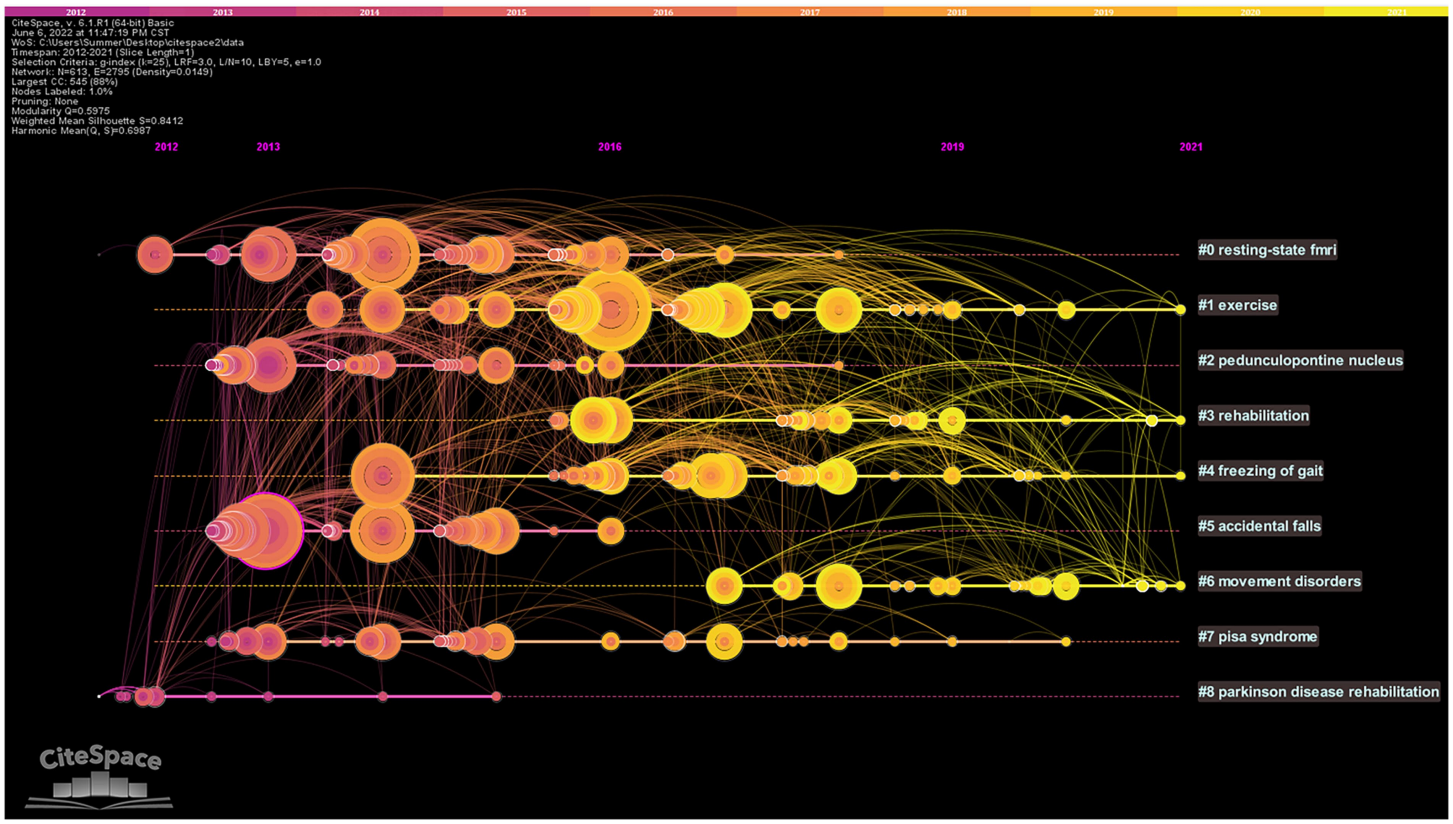
Timeline map of co-cited literature. The top of the picture represents the timeline. Nodes represent references. The nodes are distributed from left to right on the time axis, representing the time of the first publication of the cited literature from far to near. # represents different cluster labels.

Based on the co-citation literature, the timeline map of co-cited literature was obtained after labeling the clusters by extracting the noun terms from the keywords (Figure 7). The distribution of node centers from left to right on the plot represents the year when the cited document was first published from far to near, which reflects the temporal characteristics and evolutionary trends of the cited literature (Wang et al., 2021). Some study topics in the timeline map have a short duration, such as cluster (#5) accidental falls. However, some clusters are still active at present, such as (#1) exercise, (#3) rehabilitation, (#4) freezing of gait, and (#6) movement disorder, which represent the research hotspots in this field.

#### 3.5.3. Co-cited representative literature

The top 10 pieces of literature with the highest co-citation frequency are determined using co-citation frequency as the primary index (Table 3). One of the key metrics to gauge the significance of literature is the frequency of citations, which shows the academic contribution of literature in the relevant field (Cheng et al., 2021). The top 10 co-cited literature mainly include reviews (four papers; Nutt et al., 2011; Allen et al., 2013; Schoneburg et al., 2013; Abbruzzese et al., 2016), cohort studies (two papers; Kerr et al., 2010; Mancini et al., 2012), cross-sectional studies (two papers; Stebbins et al., 2013; Schlenstedt et al., 2016), and randomized controlled trials (two papers; Li et al., 2012; Curtze et al., 2015).

**Table 3:** Top 10 co-citation representative literature on postural control of PD patients among the 1,295 articles included (2011–2021).

Rank	Cited number	Title	Type	Year	Centrality	Journal	IF	Reference
1	50	Levodopa is a Double-edged sword for balance and gait in people with Parkinson’s disease	RCT	2015	0.08	Movement disorders	9.698	Curtze et al. (2015)
2	45	Predictors of future falls in Parkinson’s disease	Cohort	2010	0.01	Neurology	11.800	Kerr et al. (2010)
3	43	Tai chi and postural stability in patients with Parkinson’s disease	RCT	2012	0.12	New England Journal of Medicine	176.079	Li et al. (2012)
4	40	How to identify tremor dominant and postural instability/gait difficulty groups with the movement disorder society unified Parkinson’s disease rating scale: comparison with the unified Parkinson’s disease rating scale	Cross-S	2013	0.04	Movement Disorders	9.698	Stebbins et al. (2013)
5	36	Recurrent falls in Parkinson’s disease: a systematic review	Review	2013	0.05	Parkinson’s disease	3.170	Allen et al. (2013)
6	33	Framework for understanding balance dysfunction in Parkinson’s disease	Review	2013	0.07	Movement disorders	9.698	Schoneburg et al. (2013)
7	31	Freezing of gait: moving forward on a mysterious clinical phenomenon	Review	2011	0.04	Lancet Neurology	59.935	Nutt et al. (2011)
8	29	Postural control and freezing of gait in Parkinson’s disease	Cross-S	2016	0.03	Parkinsonism & Related Disorders	4.402	Schlenstedt et al. (2016)
9	29	Rehabilitation for Parkinson’s disease: Current outlook and future challenges	Review	2016	0.05	Parkinsonism & Related Disorders	4.402	Abbruzzese et al. (2016)
10	28	Postural sway as a marker of progression in Parkinson’s disease: a pilot longitudinal study	Cohort	2012	0.02	Gait & Posture	2.746	Mancini et al. (2012)

^*^RCT, randomized controlled trial; Cohort, cohort study; Cross-S: cross-sectional study; IF, impact factor; ^*^Review: literature review, including systematic review.

From the literature content, some researchers have studied the fall risk factors, postural control system, freezing of gait, the current outlook and future challenges of rehabilitation treatment with PD, and pointed out the relationship between freezing of gait and postural control in PD patients, which reduced their postural control ability to some extent. Other researchers suggested that using the *Movement Disorder Society Unified Parkinson’s Disease Rating Scale* (*MDS-UPDRS*) and postural swing might facilitate the distinction of different classifications and the detection of disease progression in PD patients (Stebbins et al., 2013), which provided a more sufficient theoretical basis for domestic and foreign scholars to understand PD. An RCT study by Li et al. (2012) was published in the high-impact journal *New England Journal of Medicine* (IF = 176.079) in 2012. This study focuses primarily on the effect of Tai Chi on postural instability in PD patients and proposes that tai chi training can reduce the balance disorder of PD patients and reduce the risk of falling. The literature attained a high centrality (0.12), indicating that the research finding had a significant influence on the study of postural control in PD patients and provided crucial theoretical guidance for clinicians and researchers to carry out the research.

### 3.6. Keywords analysis

#### 3.6.1. Keywords co-occurrence analysis

**Figure 8:**
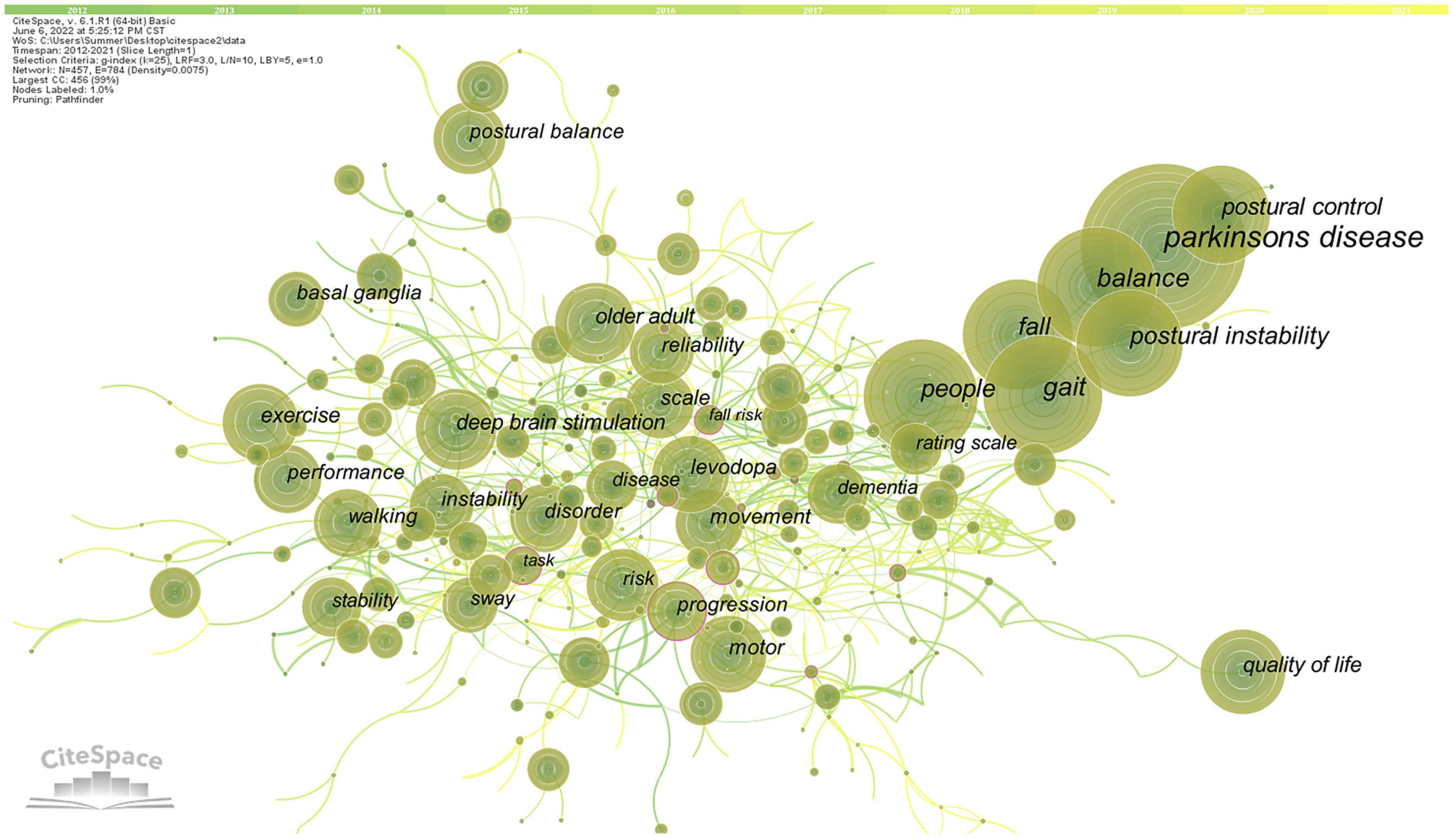
High-frequency keywords co-occurrence map of related literature. The circle in the figure represents keywords, and the larger the circle, the higher the frequency of keywords. The line between the circles represents the practice between keywords. The purple outer circle represents the centrality of keywords.

We used pathfinder, pruning sliced networks, and pruning the merged network to analyze the keywords co-occurrence network map, which obtained 457 nodes, 784 links, and a network density of 0.0075 (Figure 8). The keywords analysis reflects the research focus of an article or an author and provides a typical overview of a research trend (Liu et al., 2012). Using citation frequency as the primary observation for analysis, the top 10 high-frequency keywords cited were obtained for “Parkinson’s disease,” “balance,” “gait,” “people,” “fall,” “postural instability,” “postural control,” “exercise,” “deep brain stimulation,” and “quality of life.”

#### 3.6.2. Keywords clusters analysis

**Figure 9:**
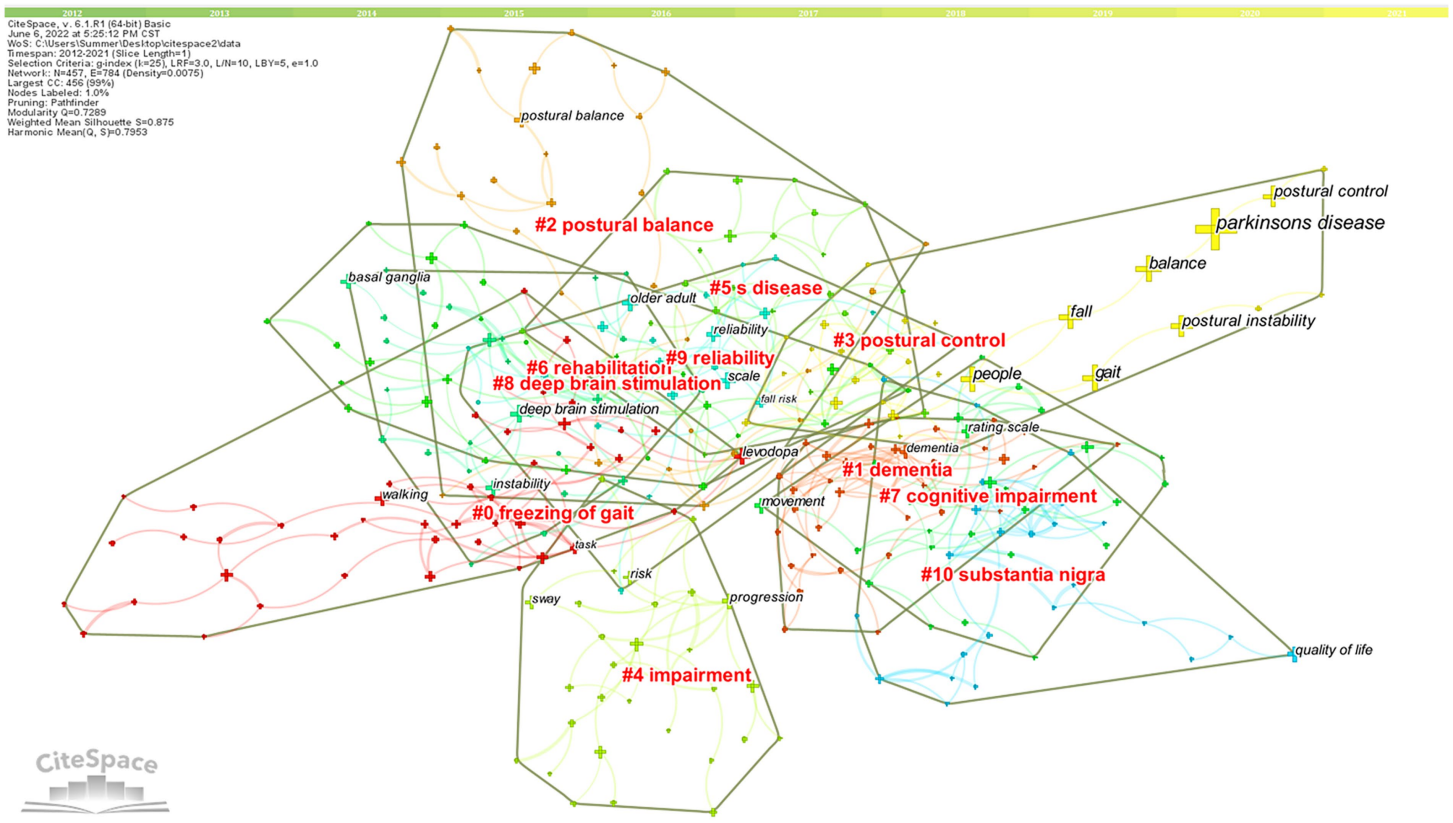
High-frequency Keywords cluster map of related literature. The nodes in the diagram represent references and the red font represents cluster labels. Nodes with the same color are located in the same cluster, which means they belong to the same cluster topic.

The keywords clusters map focuses on reflecting the structural characteristics between clusters and highlights their key nodes and important connections (Qin et al., 2020). Based on co-occurrence analysis, the LLR is used to cluster keywords to create a keywords clusters network map. The network map has a substantial clustering structure, and the structure is persuasive, according to the Q value produced by clustering keywords in this study, which is 0.7289 (>0.3), and the S value, which is 0.875 (>0.7; Figure 9). The top 11 cluster label groups (#0–10) that were examined and screened using clustering keywords represented the general research framework in the area of postural control in PD patients (Table 4). Among the 11 keywords clusters, the research topics can be divided into three categories: (#0), (#1), (#4), and (#7) are the first category, primarily involving clinical symptoms and dysfunction studies; (#2), (#3), (#5), and (#9) are the second category, mainly focusing on the research on the fall risk; (#6), (#8), and (#10) are the third category, mainly focusing on the intervention to improve the postural control ability of PD patients measure research. The number of labels in the keywords cluster is inversely proportional to the size of the cluster, with the largest cluster label being (#0) freezing of gait (46), indicating that freezing of gait has a significant impact on postural control in PD patients.

**Table 4:** List of keywords cluster labels of postural control studies in patients with PD among the 1,295 articles included (2011–2021).

Cluster-ID	Size	Silhouette	Cluster name	Cluster labels (LLR)
#0	46	0.895	Freezing of gait	Gait initiation; levodopa; anticipatory postural adjustments; and dynamic stability
#1	37	0.841	Dementia	Balance; nonmotor symptom; biomarker; and postural balance
#2	31	0.870	Postural balance	Accidental falls; exercise therapy; risk factors; and rehabilitation
#3	30	0.935	Postural control	Parkinson’s disease; postural instability; falls; and posturography
#4	30	0.875	Impairment	Risk; freezing of gait; balance dysfunction; and progression
#5	29	0.785	Parkinson’s disease	Fear of falling; clinical test; and Parkinson & apos
#6	29	0.814	Rehabilitation	Postural instability; program; muscle strength; and stroke
#7	28	0.844	Cognitive impairment	Rating scale; physical therapy; older adults; and Alzheimer’s disease
#8	27	0.897	Deep brain stimulation	Subthalamic nucleus; basal ganglia; globus pallidus; and pedunculopontine nucleus
#9	26	0.789	Reliability	Fall risk; scale; virtual reality; and postural instability gait difficulty
#10	26	0.943	Substantia Nigra	Progressive supranuclear palsy; multiple system atrophy; balance; and pedunculopontine tegmental nucleus

* LLR is Log-Likelihood Ratio.

#### 3.6.3. Keywords time zone map analysis

**Figure 10:**
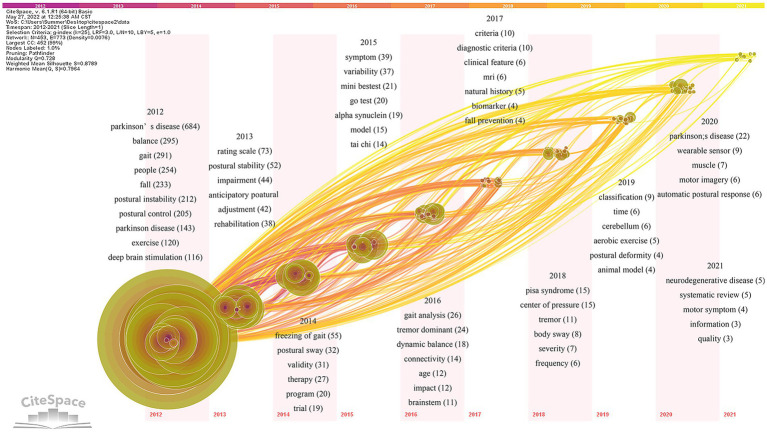
Keywords time zone map of related literature. The time zone diagram increases in the year from left to right. Using the year “1” as a time slice, keywords that first appear in the same year are aggregated in the same time zone.

Based on the keywords co-occurrence analysis, the keywords time zone map is obtained by selecting “time zone view” under the layout in the control panel (Figure 10). The keywords time zone map focuses on representing the evolution of high-frequency keywords from the time dimension, which can clearly show the update and interaction of keywords (Zhang et al., 2021). The content of research on postural control in PD patients in the last decade is mainly focused on Parkinson’s disease, covering several research hotspots, such as balance (295), gait (291), falls (233), postural instability (212), and postural control (143). Early studies on interventions to improve postural control in PD patients are highly regarded including exercise (120) and deep brain electrical stimulation (116). Research hotspots in various periods gradually started to appear as the years went on.

The specific analysis is as follows: the pathogenesis of postural control disorders in PD patients is defined by terms like α-synuclein (19), age (12), brainstem (11), biomarkers (4), cerebellum (6), and animal model (4). Keywords such as systematic evaluation (5) and randomized controlled trial (19) describe the study methods. The keywords such as scale evaluation (73), postural sway (32), Mini-Balance Evaluation System Test (Mini-BESTest; 21), Time Up and Go Test (TUGT; 20), gait analysis (26), the center of pressure (15), and wearable devices (9) define the assessment tools of postural control studies in PD patients. Dysfunction (44), anticipatory postural adjustment (42), freezing of gait (55), tremor (35), dynamic balance (18), fall prevention (4), Pisa syndrome (15), postural deformity (4), automatic postural response (6), quality of life (3), natural history (5), and muscle function (7) are hot observation feature in the field. Tai Chi(14), MRI (6), aerobic exercise (5), and motor imagery (6) may effectively improve postural control. In addition, the definition of Parkinson’s disease (76), diagnostic criteria (20), motor phenotype (22), and rehabilitation (38) are also hot elements of research in the field.

#### 3.6.4. Keywords citation burst analysis

**Figure 11:**
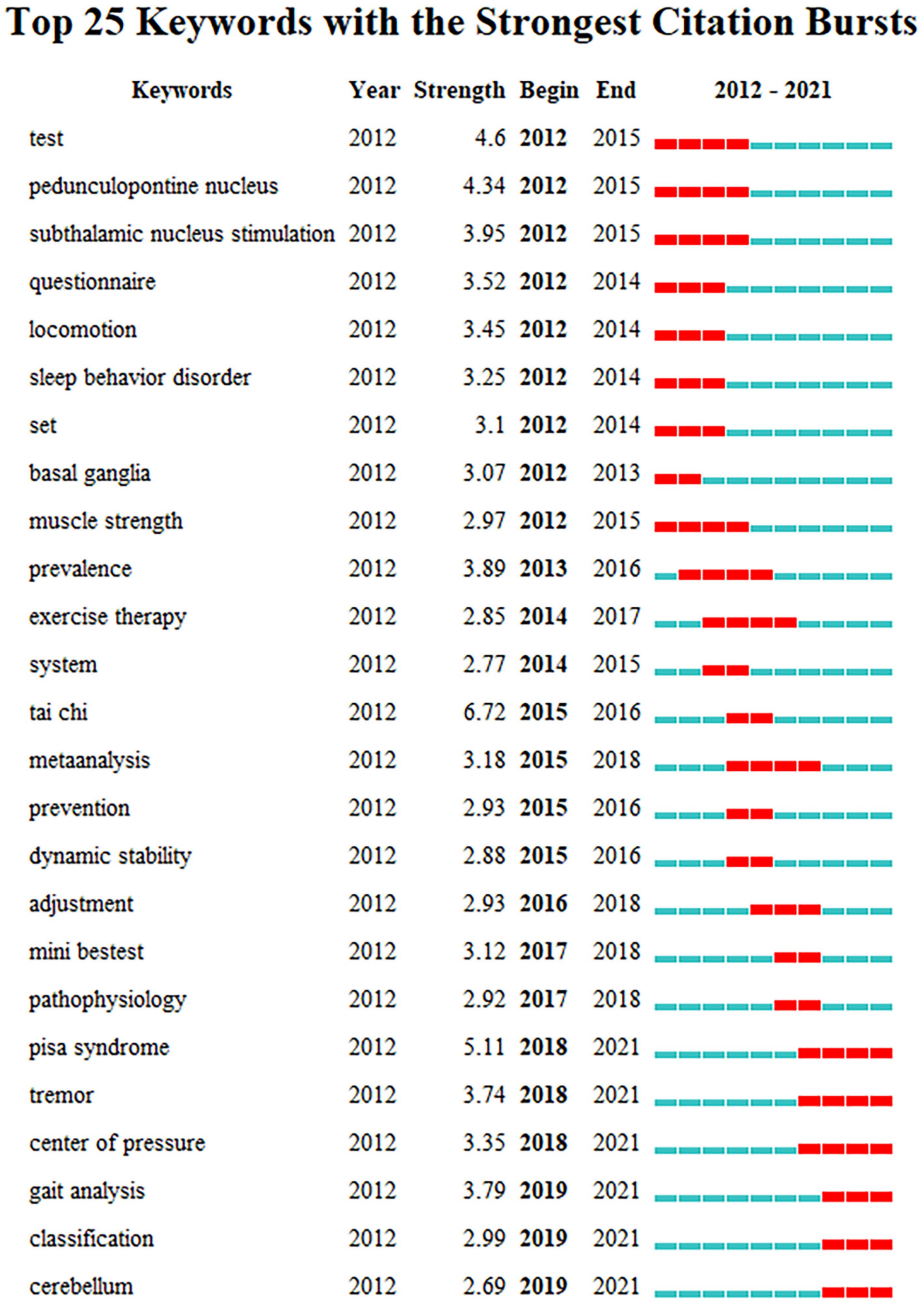
Keywords citation bursts analysis of related literature. The red line shows the time frame during which the keyword bursts were discovered, while the blue line shows the time interval.

The study of keywords citation burst can clearly reflect the research hotspots of the literature in the field at a certain period of time (Wang et al., 2020). The top 25 cited keywords were determined using keywords citation burst analysis based on keywords co-occurrence analysis (Figure 11). In the keyword burst analysis, “Begin” and “End” represent the time of the burst. “Strength” refers to the intensity of the burst, which stands for believability over time (Luo et al., 2021). The hot topics in the research sector can be discovered by using keywords with a higher burst, which signify phrases with a higher frequency of change over a specific period (Wang et al., 2019).

The keywords citation burst analysis can be divided into three phases: the first phase is from 2012 to 2015, during which time the burst keywords primarily focus on “test, pedunculopontine nucleus, thalamic floor nucleus stimulation, movement, sleep behavior disorder, basal ganglia, muscle strength.” The underlying causes of postural control impairments in PD patients are the main topic of this phase. For example, the pedunculopontine nucleus (PPN; 4.34) is a crucial component of the mesencephalic locomotor region and reticular activating system, and it is strongly connected to the basal ganglia. The PPN is directly connected to the cortical motor area to control gait and postural (Takakusaki et al., 2016), playing an important role in the pathogenesis of PD. Damage to this area may result in gait and postural control disorders (Fraix et al., 2013). The second phase is from 2015 to 2018, during which time the burst keywords primarily focus on “epidemic, movement therapy, system, tai chi, meta-analysis, prevention, dynamic stability, anticipatory postural adjustment, Mini-BESTest, and pathophysiology.” This stage mainly focuses on the techniques for assessing and treating postural stability in PD patients. “Tai Chi” (6.72) became the keyword with the highest burst intensity at this stage, indicating that Tai Chi may play an important role in improving postural control in PD patients. The third phase is from 2018 to 2021, and the primary burst keywords are “Pisa syndrome, tremor, the center of pressure, gait analysis, motor phenotype, and cerebellum.” “Pisa syndrome” (5.11) was the most burst keyword during this period, suggesting that it may be an important clinical feature for researchers to focus on when improving postural control in PD patients. In recent years, “center of pressure” and “gait analysis” have been the most popular indicators and analysis methods for assessing postural control and gait function in PD patients. The burst keyword “cerebellum” reveals that the cerebellum region may be an important neuromodulatory target for improving postural instability in PD patients in the future. The keywords of the third phase are still active now, implying new trends and directions for future research on the topic have emerged recently.

## 4. Discussion

This study used the WOSCC database to search for relevant literature in the field of postural control in PD patients from 2011 to 2021. Citespace V software was then used to visually analyze the literature contents so that we could learn more about the research status of postural control in PD patients over the past 10 years and explore research hotspots and frontiers in this field.

### 4.1. Current status of research

In the past 10 years, there has been an increase in the number of articles published each year in the international research literature on postural control in PD patients, with the biggest jump occurring between 2019 and 2020. This upward trend in article production over time suggests that postural control in PD patients has gradually gained importance as a research topic. The United States has the most publications in the area of postural control in PD patients when looking at the analysis of global research influence, and its renowned university, *Oregon Health and Science University*, has also made a significant contribution. Additionally, the USA has a significant influence thanks to its increased international collaboration. The most widely published authors on this subject, according to the visualized analysis findings of authors, are Horak FB, Bloem BR, and Mancini Martina. The use of wearable inertial sensor devices to monitor postural sway (Mancini et al., 2011, 2012), balance (Hasegawa et al., 2019), gait (de Lima-Pardini et al., 2017), and disease progression (Zampieri et al., 2010; Mancini and Horak, 2016) in PD patients is a key component of the research direction of *the Oregon Health and Science University* team. Professor Bloem BR’s research interests in this area are mostly connected to postural control in PD patients, including the freezing of gait (Bekkers et al., 2018a,b, 2020), assessment tests (Geurts et al., 2011; Bloem et al., 2016; Silva de Lima et al., 2020), and postural control mechanisms (Nonnekes et al., 2013).

### 4.2. Important research findings

According to the citation frequency analysis of literature, the paper published in the journal “*Movement Disorders”* by Curtze et al. (2015) obtained the most citations. Levodopa medicines have a dual effect on balance and gait in PD patients, according to the study, which noted that while levodopa helps control balance and gait, numerous movement abnormalities may be either directly or indirectly induced by levodopa. The second most frequently cited literature is a prospective cohort study published in Neurology by Kerr et al. (2010), which showed that the combination of both disease-specific and balance- and activity-related measurements are important for predicting falls in PD patients. A combination of the *Unified Parkinson’s Disease Rating Scale* (UPDRS) total score, FOG total score, Tinetti total score, the occurrence of symptomatic postural orthostasis, and extent of postural sway in the anterior–posterior direction is the multivariate model that gave the best sensitivity (78%) and specificity (84%) results.

Furthermore, Li et al. (2012) evaluated the effects of three different exercise modalities (tai chi, resistance training, and stretching) on postural stability in PD patients and asserted that tai chi might enhance balance dysfunction and reduce the frequency of falls in people with the condition. When the study was finally published, it attracted a lot of citations in the *New England Journal of Medicine*. The three mentioned research findings are significant findings in the field of postural control research with PD, as they were conducted from the perspectives of influencing factors, assessment tools, and interventions. Such perspectives are highly regarded and innovative in this research field.

### 4.3. Research hotspots and frontiers

Based on the visualized findings, including the co-cited literature timeline map, keywords time zone map, and keywords burst map, it could reflect the research trends and popular topics in the field. In recent 10 years, keywords such as “neurodegenerative diseases,” “motor symptoms,” “α-synuclein,” and “biomarkers” have defined the new approach to PD diagnosis and mechanism research. PD is the second most prevalent neurodegenerative disease after Alzheimer’s disease. Its etiology is complex, and α-synuclein is the key component of the pathogenesis of the disease (Rocha et al., 2018). Since the clinical diagnosis of PD is challenging, finding early biomarkers and accurate diagnostics for PD is crucial for clinical trials and treatment of PD (Atik et al., 2016; Fayyad et al., 2019). According to research, α-synuclein aggregates in cerebrospinal fluid (CSF) can be used as a biochemical diagnostic for the differential diagnosis of PD and are crucial for distinguishing PD from multisystem atrophy (Shahnawaz et al., 2020). In light of the crucial role that α-synuclein plays in the diagnosis of PD, α-synuclein has been discovered as a biomarker of PD with a crucial role in tracking the course of PD disease (Atik et al., 2016). Studies have noted a high correlation between CSF and plasma/serum neurofilament light chain (NfL; Bacioglu et al., 2016). Blood-based biomarkers, however, might be better than cerebrospinal fluid biomarkers due to the difficulty of cerebrospinal fluid sampling (Lin et al., 2019). NfL is a marker of axonal injury (Oosterveld et al., 2020). A valid biomarker of PD disease progression, plasma NfL levels are significantly increased in PD patients with advanced disease and dementia, demonstrating that NfL levels correlate with disease severity as well as motor and cognitive function in PD patients (Lin et al., 2019). To determine whether the addition of CSF or serum NfL to alpha-synuclein contributes to the diagnosis of PD, Oosterveld et al. (2020) combined CSF neurofilament, phosphorylated−/total alpha-synuclein, and oligomeric−/total alpha-synuclein to obtain the CSF biomarker battery, which was considered as the best biomarker model to differentiate PD patients from healthy individuals.

“Posture deformity,” “freezing of gait,” and “falls” related to posture control of PD patients have also become the frontier contents in the research field. Postural deformity, such as camptocormia, antecollis, Pisa syndrome, and scoliosis, are frequent and disabling complications in PD patients (Doherty et al., 2011). The different manifestations of postural deformities are the result of the interaction of many complex factors, mainly including muscle rigidity, axial dystonia, myopathy, impaired central proprioception causing changes in body structure, degenerative changes in the spine and soft tissues, etc. (Doherty et al., 2011). Pisa syndrome (PS), for instance, is an abnormal posture in which the trunk is laterally flexed more than 10°. The underlying causes may be muscular imbalance and compensatory posture brought on by muscular rigidity, myopathy, and soft tissue changes (Doherty et al., 2011). Compared to PD patients without PS, PD patients with PS have increased body sway, and postural alignment difficulty, and show poorer postural control (Geroin et al., 2015). Both freezing of gait and postural control impairment are late symptoms of PD patients and are strongly associated with fall risk. More than 50% of patients with severe PD are affected by the freezing of gait (Borzì et al., 2021). According to studies, PD patients with FOG (PD + FOG) have poorer postural control compared to PD patients without FOG (PD − FOG) and the general population (Vervoort et al., 2016; Coelho et al., 2021). Differences in postural control are particularly evident when patients perform the sit-walk task (Mezzarobba et al., 2018).

The selection of appropriate and accurate gait and balance assessment methods is essential to facilitate the effective treatment of PD patients. Numerous clinical instruments, such as the Berg Balance Scale (BBS), Tinetti, Mini-Balance Evaluation Systems Test (Mini-BESTest), and TUGT, are used to evaluate balance and postural control in PD patients. In general, assessment scales can reflect various aspects of postural control and has the characteristics of low cost, short time consumption, and method simply. However, the scales are subjective and the measurement results are not sufficiently detailed, and clinicians or researchers are prone to getting mixed up with personal subjective factors when evaluating assessments. With the advancement of experimental techniques and scientific tools, more researchers are using “posturography,” “wearable devices,” “gait analysis,” and “center of pressure” to track the postural control and gait of PD patients. By means of posturography, it has been observed that in the early stages of Parkinson’s disease, patients have a decrease in the limit of stability (LOS) area and an increase in postural sway, and these conditions gradually deteriorate as the disease progresses (Doná et al., 2016). Wearable devices can be installed in the neck, waist, back, lower limbs, and other body parts, and have been widely utilized for the self-detection of PD patients in many aspects. Wearable sensors can not only objectively measure the effects of exercise interventions on motor function, and provide support for the measurement of gait and sit-to-stand tasks (Flood et al., 2020), but also detect subtle changes in postural instability and fall risk of PD patients (Stack et al., 2018).

Axial symptoms such as gait disturbances, postural instability, and balance dysfunction have become major treatment challenges in patients with advanced PD (Amano et al., 2013; Potter-Nerger and Volkmann, 2013). Deep brain stimulation (DBS) is a well-established treatment for improving dyskinesia and tremor in PD (Spindler et al., 2022). Studies have shown that DBS targeting the subthalamic nucleus (STN) and Globus pallidus pars interna (GPi) can improve the postural control and gait parameters of PD patients in quiet standing (Collomb-Clerc and Welter, 2015), but no efficacy has been found for freezing of gait (Potter-Nerger and Volkmann, 2013). Following a comparison of the short- and long-term effects of DBS on gait and related major symptoms in PD patients, Brozova et al. (2021) found that short-term DBS treatment was similar to dopamine medication in improving gait and postural instability, whereas gait improvement was significantly reduced after long-term or high-frequency DBS treatment, even with deleterious effects on gait function. Noninvasive brain stimulation (NIBS) is a safe and effective treatment modality (Liang et al., 2020). The most widely used non-invasive brain stimulation techniques are repetitive transcranial magnetic stimulation (rTMS) and transcranial direct current stimulation (tDCS), both of which have been applied in the rehabilitation of balance and postural control with PD. It has been demonstrated that using tDCS to target the primary motor cortex (M1) and motor cortex leg areas improves perturbation response and postural stability (Beretta et al., 2020; Liang et al., 2020), but the choice of stimulation target can have a large variation on the treatment effect. The cerebellar region is functional tissue that play an important role in anticipatory postural adjustment (APA) during gait initiation, as well as in the coupling of lower limb muscle activation patterns (Richard et al., 2017). Based on resting-state functional magnetic resonance imaging (fMRI), a researcher has discovered that increased functional connectivity in the cerebellar structures of PD patients may support the activation of additional brain structures as a compensatory mechanism to help patients regain gait function. The cerebellar region may be a future neuromodulatory region for the treatment of postural control in PD patients (Kaut et al., 2020).

Rehabilitation interventions such as “Tai Chi,” “exercise,” “aerobic exercise,” and “motor imagery” have become research hotspots in recent 10 years. Tai chi, a popular, secure, and efficient form of exercise, is crucial for improving postural control in PD patients (Ni et al., 2014). Long-term Tai Chi training can improve brain network function, improve amino acid metabolism, energy metabolism, and neurotransmitter metabolism, as well as reduce the inflammatory response and decrease dopaminergic degeneration susceptibility (Li et al., 2022). Furthermore, it has significant effects on improving gait, balance, and motor function while decreasing the risk of falls in PD patients (Yang et al., 2014; Scianni, 2015; Zhong et al., 2020b). Regular aerobic exercise has been shown in studies to improve postural control when compared to sedentary people (Catalán Edo et al., 2021). de Oliveira et al. (2021) conducted a systematic review of the literature on the effects of aerobic exercise on functional capacity and quality of life in patients with mild to moderate Parkinson’s disease, which discovered that aerobic exercise improved gait, mobility, and lower extremity muscle strength. Exercise interventions such as treadmill gait training, Nordic walking, brisk walking, and dance, have all been proven to be effective in improving the balance function and motor symptoms of PD patients, and may delay the progression of the disease (Mak and Wong-Yu, 2019). Motor imagery (MI) is a mental rehearsal method in which movements are imagined but not performed (Kim et al., 2018). MI is a cost-effective, low-risk alternative motor therapy modality. MI can improve motor skills by enhancing the proprioceptive signals that patients normally generate during movements, thereby enhancing the learning of new tasks and improving motor performance(Abbruzzese et al., 2016). According to research, a combination of physical exercise and motor imagery can effectively alleviate motor latency in PD patients (Tamir et al., 2007). Furthermore, incorporating motor imagery into dual-task gait or balance training may promote specific functional reorganization of brain regions of motor control and executive ability or attention, as well as have a longer-lasting effect on dual-task activity and balance in PD patients with postural instability and gait impairment (Sarasso et al., 2021).

### 4.4. Limitations

In this paper, Citespace 6.1R1 software is used to visually analyze the related literature on posture control of patients with PD over the past last decade in the database of Web of Science Core Collection, but the following limitations still exist: firstly, the economic power and population size of different countries vary, which will also influence the research development of different countries in this research field and may introduce bias; secondly, only English literature in the Web of Science core collection database is included for visualized analysis, and future research can combine the literature from CNKI, Scopus, and other databases; thirdly, the visualization tool is relatively simple. In the future, we can conduct VOSviewer and Gephi software to provide a more comprehensive and clearer theoretical reference for the study of postural control in PD patients.

## 5. Conclusion

This study is the first to use citespace V software to visualize and analyze the literature in the field of postural control in PD patients from 2011 to 2021 in the web of science core collection database, which can reflect the current research status, research hotspots, and future development trends in this field with a more intuitive, efficient and scientific way.

The results of this study indicate that the annual volume of postural control in PD patients tends to increase. The United States and its famous university (Oregon Health and Science University) are the main country and institution, and Horak FB. from this university is the main author in this research field. In recent 10 years, the literature in the field of postural control in PD patients mainly focuses on the review and clinical research of fall risk prediction and factors, postural control system, freezing of gait, drugs, and sports rehabilitation. With further research, exploring the research of early biomarkers and mechanisms of PD disease and using wearable devices or gait analysis methods to monitor and assess postural control of PD patients has led to research into the molecular/biology/genetics field and the health/rehabilitation/psychology/social field. Based on the dual-map overlay of journals, *Movement Disorders* is the most frequently cited journal, and future research will focus on the neurology/sports/ophthalmology field. For example, neuromodulation therapy targeting the cerebellum site, using training methods such as Tai Chi, aerobic exercise, and motor imagery to improve the postural control of PD patients will be the next development direction in the field of postural control in PD patients.

## Author contributions

YL designed the manuscript, collected and analyzed the data, drafted the manuscript, and finished the translation. XW and WG designed the manuscript. XW and C-JL revised the text of the manuscript. J-JZ provided revisions and critically reviewed the content of the manuscript. All authors contributed to the article and approved the submitted version.

## Funding

This work was supported by the National key R&D program of China (2020YFC2008702), Shanghai Clinical Research Center for Rehabilitation Medine (21MC1930200), and Special Project of Medical Innovation Research of Shanghai “Science and Technology Innovation Action Plan” (22Y31900200).

## Conflict of interest

The authors declare that the research was conducted in the absence of any commercial or financial relationships that could be construed as a potential conflict of interest.

## Publisher’s note

All claims expressed in this article are solely those of the authors and do not necessarily represent those of their affiliated organizations, or those of the publisher, the editors and the reviewers. Any product that may be evaluated in this article, or claim that may be made by its manufacturer, is not guaranteed or endorsed by the publisher.
